# Impact of an intervention to improve pit latrine emptying practices in low income urban neighborhoods of Maputo, Mozambique

**DOI:** 10.1016/j.ijheh.2020.113480

**Published:** 2020-05

**Authors:** Drew Capone, Helen Buxton, Oliver Cumming, Robert Dreibelbis, Jackie Knee, Rassul Nalá, Ian Ross, Joe Brown

**Affiliations:** a Civil and Environmental Engineering, Georgia Institute of Technology, Atlanta, GA, USA; b Department of Disease Control, London School of Hygiene and Tropical Medicine, London, UK; c Ministério da Saúde, Instituto Nacional de Saúde Maputo, Maputo, Mozambique

**Keywords:** Fecal sludge management, Hygienic pit latrine emptying, On-site sanitation, pour-flush latrines, Fecal sludge transport, Sanitation intervention

## Abstract

Safe fecal sludge management (FSM) – the hygienic emptying, transport, and treatment for reuse or disposal of fecal sludge – is an essential part of safely managed sanitation, especially in towns and cities in low- and middle-income countries with limited sewer coverage. The need for safe and affordable FSM services has become more acute as cities grow and densify. Hygienic pit-emptying uses equipment that limits direct human exposure with fecal sludge and hygienic transport conveys fecal sludge offsite for treatment. We evaluated whether a program of on-site sanitation infrastructure upgrades and FSM capacity development in urban Maputo, Mozambique resulted in more hygienic pit-emptying and safe transportation of fecal sludge. We compared reported emptying practices among multi-household compounds receiving sanitation upgrades with control compounds, both from the Maputo Sanitation (MapSan) trial at 24–36 months after the intervention. Intervention compounds (comprising 1–40 households, median = 3) received a subsidized pour-flush latrine to septic tank system that replaced an existing shared latrine; control compounds continued using existing shared latrines. We surveyed compound residents and analyzed available municipal data on FSM in the city. Due to the recent construction of the intervention, emptying was more frequent in control compounds: 5.6% (15/270) of intervention compounds and 30% (74/247) of controls had emptied their on-site sanitation system in the previous year. Among those compounds which had emptied a sanitation facility in the previous year, intervention compounds were 3.8 (95% CI: 1.4, 10) times more likely to have to done so hygienically. Results suggest that the construction of subsidized pour-flush sanitation systems increased hygienic emptying of fecal sludge in this setting. Further gains in hygienic emptying in urban Maputo may be limited by affordability and physical accessibility.

## 1 Background

The United Nations Sustainable Development Goal (SDG) 6 calls for universal access to “safely managed” sanitation by 2030, defined as the “use of improved facilities that are not shared with other households and where excreta are safely disposed of *in situ* or transported and treated offsite” (UNICEF & WHO, 2017). Piped sewerage and centralized wastewater treatment only serves an estimated 316 million people in cities of LMICs (Berendes et al., 2017). For many cities in LMICs, expansion of networked sewerage to growing populations can be cost-prohibitive (Dodane et al., 2012) and some cities struggle to adequately maintain existing sewer infrastructure (Dodane et al., 2012; van Esch and van Ramshorst, 2014). Expanding networked sewerage to unplanned settlements is often complicated by complex or unclear land tenure, high population and housing densities limiting access for construction, and an absence of city planning and basic infrastructure (Un-Habitat, 2003). In addition, reticulated systems, reliant on a large and consistently reliable supply of water, may not be sustainable nor desirable (Mafuta et al., 2011).

**Fig. A.1:**
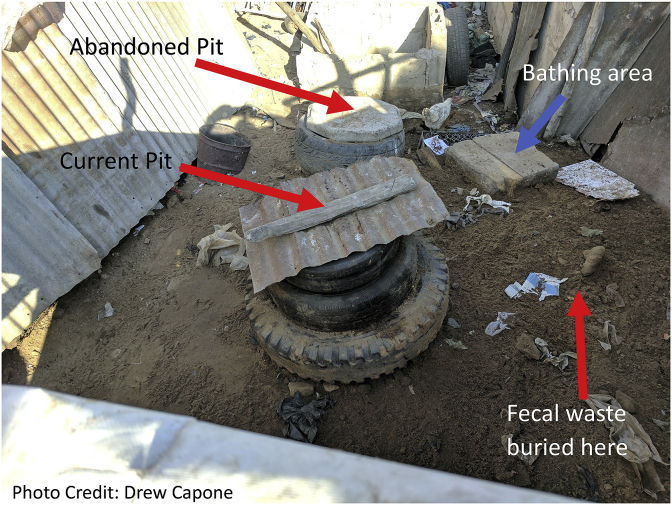
Bathroom in low-income urban Maputo.

When on-site sanitation systems—such as pit latrines or septic tanks—fill, fecal sludge must be either safely sequestered *in situ* or must be hygienically emptied, safely transported, and adequately treated for reuse or disposal. As space becomes increasingly limited in densifying urban and peri-urban communities where on-site systems predominate, the common practice of covering and abandoning pits once full becomes unworkable (Fig. A.1). Decreasing space and the construction of more permanent latrine superstructures necessitate emptying as part of a safely managed fecal sludge management (FSM) service chain (Peal et al., 2014a). Hygienic emptying and transport of fecal wastes to a treatment plant are necessary to reduce exposures to fecal-oral pathogens, as repeated exposures to fecal-oral pathogens are associated with negative impacts on child health and survival (Troeger et al., 2018; World Health Organization, 2018). Achieving SDG sanitation targets will therefore require providing safe and hygienic FSM services to at least 1.8 billion people who rely on on-site sanitation technologies and lack access to these services (Berendes et al., 2017; Peal et al., 2014b; Scott et al., 2018).

The Maputo metropolitan area contains 2.7 million people (Hanlon, 2018) but only 136,000 people are served by a sewer connection and most of the wastewater from the sewers discharges untreated into Maputo Bay (Ministry of Public Works, 2015; van Esch and van Ramshorst, 2014). Of the unsewered population, 36% of households use dry pit latrines and 64% use pour-flush latrines with disposal to pits or septic tanks (Hawkins and Muximpua, 2015). Most households have a private on-site sanitation facility; 16% of on-site sanitation facilities are shared by two or more households (Bauerl et al., 2016). Maputo has no designated treatment facility for fecal sludge. Instead, fecal sludge is discharged to anaerobic ponds at a nearby wastewater treatment plant (WWTP) according to municipal by-laws (Assembleia Municipal de Maputo, 2017; Bauerl et al., 2016).

Pit-emptying businesses and organizations can pay an annual fee to formally register with Maputo Municipal Council (US$ 67–167) and a monthly fee to dispose fecal sludge at the WWTP ($25-$75) (Assembleia Municipal de Maputo, 2017). Fines for illegal dumping of fecal sludge range from $100-$167 USD, but it is unclear how commonly fines are imposed. Unhygienic informal pit-emptying is illegal in Maputo but remains common across the low-income areas. An estimated 60% of emptying was performed by unhygienic emptiers in 2016 (Bauerl et al., 2016). In 2013 it was estimated that 100% of fecal sludge from unhygienic emptying was disposed illegally in or near customers’ yards, while 25% of fecal sludge from more hygienic emptiers was disposed illegally (Bauerl et al., 2016; Peal et al., 2015).

Pit-emptying practices can be categorized as hygienic or not based on plausible exposure risks to the community and those engaged in emptying and transportation (Jenkins et al., 2015; Thye et al., 2011). Unhygienic pit-emptying typically uses manual equipment, such as buckets and shovels, and is often performed at night due to social stigma and the intense smell produced (Chowdhry and Kone, 2012); fecal sludge is often buried in the customer's yard or dumped nearby in a drain or ditch, which may contribute to the transmission of enteric pathogens through well-understood pathways (Hawkins and Muximpua, 2015; Jenkins et al., 2015; Peal et al., 2015; Thye et al., 2011; Wagner and Lanoix, 1958). Household members or manual laborers from the local community often perform unhygienic emptying.

Hygienic pit-emptying typically uses mechanized equipment (e.g. a trash pump or vacuum tanker) or manual pumps (e.g., a Gulper) (Clasen et al., 2014; GOAL, 2016; Jenkins et al., 2015; Strande et al., 2014) together with personal protective equipment (e.g. gloves, mask, boots, and work uniform). Mechanical emptying is hygienically preferable to manual emptying as it lessens the risk of contact with fecal sludge by both emptiers and residents, but some fecal sludge may still spread into the environment due to the aerosolization of microbes, the poor condition of hoses used to pump fecal sludge, dismantling of hoses after emptying, and inadequate cleaning of equipment (Farling et al., 2019; Williams and Overbo, 2015). Hygienic emptying is often performed by businesses and community-based organizations that may be registered with a local municipality to provide emptying services. These hygienic emptiers are more likely to transport fecal sludge to a treatment plant than to bury or dump it near the customer's home, due to reputational risk or government regulations. Hygienic pit-emptying is an important first step in the FSM service chain.

In high-density unplanned settlements where space is unavailable to allow the covering and abandonment of full pits, the transport of fecal sludge can be categorized as hygienic or not by considering where the fecal sludge is deposited. In such settings, fecal sludge emptied and dumped on-site may pose greater pathogen transmission risks than excreta which is transported to a treatment plant (Hawkins and Muximpua, 2015; Peal et al., 2015; World Health Organization, 2018). However, it is difficult for customers to know where their waste is deposited once it leaves their property. Tipping fees, traffic, and long transport times may discourage emptiers from disposing of fecal sludge at treatment plants (Mbéguéré et al., 2010; Strande et al., 2014).

The objectives of our cross-sectional study in the *Nhlamankulu* and *KaMaxaquene* Districts of Maputo were to: (1) evaluate the effect of an on-site sanitation and FSM strengthening intervention on increased hygienic pit-emptying and transportation of fecal sludge and (2) identify key remaining barriers to the uptake of safe FSM practices.

### 1.1 Maputo Sanitation Project

The Maputo Sanitation Project aimed to improve sanitation conditions and FSM for the residents living in the 11 low-income neighborhoods (*bairros*) of the *Nhlamankulu* District and 5 neighborhoods of the *KaMaxaquene* District in Maputo, Mozambique (Water and Sanitation for the Urban Poor, 2018). The project used an approach with three components: (1) construction of privately shared on-site sanitation infrastructure, (2) support for community based organizations (CBOs) and commercial micro-enterprises to provide desludging services and (3) community level sanitation and hygiene promotion (Water and Sanitation for the Urban Poor, 2018). Component one targeted specific compounds (household clusters), while components two and three targeted neighborhoods in the *Nlhamakulu* and *KaMaxaquene* Districts. The program was funded by the Japanese Social Development Fund (JSDF); Water and Sanitation for the Urban Poor (WSUP) was responsible for implementing the sanitation infrastructure component, development of desludging services was a joint venture between WSUP and the Water and Sanitation Program (WSP) of the World Bank, and WSUP was responsible for the community level sanitation and hygiene promotion (Hawkins and Muximpua, 2015).

Component one, the construction of sanitation infrastructure, consisted of subsidized provision of pour-flush toilets (to septic tank with a drain field) shared by multiple households in compounds (Text A.1). Compounds with approximately 15–20 people received a shared latrine (SL) and generally compounds with ≥21 people received a community sanitation block (CSB). Compound residents were expected to contribute about 8–10% of the construction cost (compounds contributed on average $97 for a CSB and $64 for a SL per compound), but operation and maintenance costs, including pit-emptying, were not subsidized (Water and Sanitation for the Urban Poor, 2018). High-water table areas were excluded from receiving sanitation infrastructure to prevent water infiltration into the system sub-structure. Additionally, intervention systems were designed with the intention that future emptying would be performed hygienically with mechanized equipment every two years; access by a vacuum truck was a site criterion for community sanitation blocks (≤60 m from a truck-accessible road) but was not considered for placement of the shared latrines. The Maputo Sanitation (MapSan) trial was a controlled, before-and-after trial of the Maputo Sanitation Project component one that assessed the impact of the intervention on enteric infections and other health outcomes in children (Brown et al., 2015; Knee et al., 2018).

Component two—the development of hygienic emptying organizations—aimed to establish enterprises capable of delivering hygienic services at commercially viable rates. WSP identified eight members of a national waste management association operating in or near the *Nhlamankulu* and *KaMaxaquene* Districts and focused on providing the capacity to perform hygienic pit-emptying in addition to their existing services (typically weekly trash pick-up) (Water and Sanitation for the Urban Poor, 2018). Capacity building took the form of technical assistance, provision of emptying equipment, monitoring and evaluation of FSM services, a marketing program to promote hygienic emptying (e.g. television ads and flyers), technical development in the form of training to use the provided equipment for hygienic emptying, and training in business management.

Component three aimed to develop and support community-level sanitation hygiene promotion and monitoring activities through engagement with appointed local government officials (*chefes de quarteirão*) and community members (Hawkins and Muximpua, 2015; Water and Sanitation for the Urban Poor, 2018). Workshops were held with local stakeholders and institutions to build capacity and establish monitoring systems. Sanitation promotion activities were implemented that focused on encouraging households to improve their on-site sanitation systems (Hawkins and Muximpua, 2015). We did not evaluate component three as it fell outside the scope of this study, only occurred three neighborhoods, and its results are reported elsewhere (Hawkins and Muximpua, 2015).

## 2 Materials and methods

### 2.1 Survey groups

We conducted surveys of on-site sanitation, revealed preferences of previous pit-emptying activities, and stated preferences for future pit-emptying activities. We trained enumerators to conduct the interviews through a two-day facilitated workshop and during one week of survey piloting in December 2017 and an additional two days of survey piloting in April 2018. All questionnaires were administered in Portuguese or the local language, Changana, as requested by the respondent. Enumerators verified sanitation infrastructure by direct observation and recorded each latrine's characteristics with illustrative photographs. Our sample frame included three primary respondents: caregivers of children enrolled in the MapSan trial; an additional respondent from the same compound who was not previously enrolled in the MapSan trial (Text A.2), and compound leaders. Our sampling strategy was intended to maximize diversity among the respondents (Text A.2).

In July 2018 we met with the Maputo Municipal Council's Department of Water and Sanitation (DAS). They provided us with a list of all registered emptying businesses in Maputo and their log of visits by the businesses to the Infulene WWTP to dispose of fecal sludge during the months of August, September, October, and November in 2017, and January and February 2018. DAS did not have digitized data available for December 2017 and we excluded this data from our analysis. We conducted all surveys and interviews from April–August 2018.

### 2.2 Data analysis

We analyzed data in R version 3.5.0 (R Foundation for Statistical Computing, Vienna, Austria). To account for clustering at the compound level we used generalized estimating equations (GEE) with the “exchangeable” correlation structure and a Poisson (log) distribution for calculation of adjusted risk ratios (aRR) (Carey, 2015; Islam et al., 2018; Liang and Zeger, 1986). For Poisson regression modelling we decided *a priori* to control for household wealth, the number of people who used the respondent's on-site sanitation system as their primary sanitation facility, and the respondent's gender and age as potential confounders (Fuhrimann et al., 2016; Niwagaba et al., 2014; Peat, 2002). Due to a small sample size for the intervention and the subset of control compounds with pour flush technology that had emptied their on-site sanitation system in the previous year, we used the non-parametric *Somer's d* test to determine the association of hygienic emptying with intervention status (Somers, 1962).

We calculated household wealth using eight of the ten inputs from the Simple Poverty Scorecard for Mozambique (Schreiner, 2013). We excluded number of beds (limited data) and sanitation (Rheingans et al., 2014) from our calculation of household wealth. We did not calculate a household wealth score for compound leaders and instead used the average wealth scores of the other household respondents inside the same compound to adjust for wealth (Text A.3).

Recognizing that interviews with one to three individuals at each compound had the potential to skew survey response data, we report data from all survey respondents for stated preference questions (e.g. future intentions about emptying) and a single priority-based respondent from each compound for revealed preference questions (e.g. emptied an on-site sanitation system in the previous year). To select a single respondent to represent each compound we assigned priority based on an assumption of which respondent type would most likely have access to accurate information. Therefore, for the revealed preference questions, we first analyzed the response from the compound leader, and, if no compound leader was interviewed, we used the response from the MapSan trial respondent. If neither a compound leader nor a MapSan trial respondent was interviewed, we used the response of the non-MapSan trial respondent as the compound response.

### 2.3 Ethical approvals

Before conducting a survey with a respondent, we obtained written informed consent. The study protocols were approved by the Comité Nacional de Bioética para a Saúde (CNBS), Ministério da Saúde (333/CNBS/14, 81/CNBS/18), the Ethics Committee of the London School of Hygiene and Tropical Medicine (Reference # 8345) and the Institutional Review Board of the Georgia Institute of Technology (Protocol #H15160, #H18027). The associated MapSan trial has been registered at ClinicalTrials.gov (NCT02362932).

## 3 Results

### 3.1 Respondent characteristics

We visited 403 MapSan households, of which 399 (99%) consented, and 386 non-MapSan households, of which 378 consented (98%). We visited an additional 63 households which did not meet our eligibility requirements and were not included in this study (Text A.4). The median amount of time respondents lived in their home was 15 years and the mean was 18 years. Compounds contained an average 4.3 families, 16 people, and 2.4 children under the age of five. We recruited 300 compound leaders from 149 control compounds and 151 intervention compounds, of whom 295 (98%) consented to participate.

### 3.2 Household and compound leader interviews

Respondents from intervention compounds primarily reported using shared latrines (78%) with the remainder using community sanitation blocks (22%). Respondents from control compounds primarily reported using pit latrines with (34%) or without a slab (29%); other control respondents reported using a septic tank or pour flush to an underground pit (30%) or above ground pit (7%). The quality of the on-site sanitation structure was generally better for intervention compared with control compounds, as measured by observable characteristics including building integrity and cleanliness. Enumerators observed that the slabs/floors of intervention systems were 1.6 (95% CI: 1.5, 1.8) times more likely to be in good condition with no cracks, holes, or visual structural defects compared to control systems.

Intervention household respondents more often reported a water tap inside the compound (68%) than control household respondents (57%) (Table A1). Control household respondents who reported having pour-flush sanitation more often reported a water tap inside the compound (69%) compared to those who did not report having pour-flush sanitation (50%). Both intervention and control household respondents most frequently reported running water was available for 4–6 hours a day (Table A1).

Emptying of an on-site sanitation system in the previous year was less common in intervention compounds; 5.6% (15/270) of intervention compounds reported emptying in the previous year compared to 30% (74/247) of control compounds (Table 1). Most intervention compounds that reported emptying in the previous year were shared latrines (11/15) and some were community sanitation blocks (4/15). A one-quartile increase in household wealth was not significantly associated with previous year emptying at either intervention (aRR: 0.99, 95% CI: 0.73, 1.3) or control compounds (aRR: 1.1, 95% CI: 0.57, 2.2).

**Table 1:** Responses to revealed preference survey questions from all respondents, 24–36 months following the intervention.

	Priority Compound Respondent			Household Respondents^a^		Compound Leader	
	**Control (n)**	**Intervention (n)**	**aRR (95% CI)**	Control (n)	Intervention (n)	**Control (n)**	**Intervention (n)**
**Compound emptied an on-site sanitation system in the previous year**	30% (74/247)	5.6% (15/270)	0.17 (0.09, 0.29)	29% (108/378)	7.0% (28/399)	31% (40/129)	7.0% (10/143)
**Compound constructed** ≥ **1 new on-site sanitation system(s) in the previous 3 years (excluding the intervention systems)**	34% (84/247)	15% (41/270)	0.39 (0.26, 0.58)	30% (111/378)	13% (51/399)	33% (42/129)	16% (23/143)
**Any on-site sanitation system in the compound flooded in the previous year**	7.3% (18/247)	4.8% (13/270)	0.71 (0.33, 1.5)	7.9% (30/378)	3.0% (12/399)	7.8% (10/129)	7.0% (10/143)
Responses to revealed preference questions from the subset of compounds that emptied an on-site sanitation system in the previous year							
**Hygienic emptier was used for emptying in the previous year**	14% (10/74)	73% (11/15)	3.8 (1.4, 10.0)	18% (19/108)	54% (15/28)	13% (5/40)	70% (7/10)
**Pit was emptied mechanically in the previous year**	9% (7/74)	67% (10/15)	4.5 (1.5, 14)	14% (15/108)	43% (12/28)	10% (4/40)	70% (7/10)
**Emptied fecal sludge taken to WWTP or transported from the compound to an unknown location (e.g. not buried on-site, not buried outside the compound, or respondent was unsure)**	3% (2/74)	40% (6/15)	30 (3.3, 270)	8% (9/108)	18% (5/28)	2.5% (1/40)	50% (5/10)
**Compound used an intervention pit-emptier (from component two) for emptying in the previous year**	0% (0/74)	20% (3/15)		0% (0/108)	21% (6/28)	0% (0/40)	30% (3/10)

a There were up to two household respondents per compound.

Among compounds where an on-site sanitation system was emptied in the previous year, hygienic emptying was more common at intervention compounds; 14% (10/74) of control compounds reported using a hygienic emptier compared to 73% (11/15) of intervention compounds (Table 1). All community sanitation blocks (4/4) and most shared latrines (7/11) which were emptied in the previous year used a hygienic emptier. No control compound reported using a pit-emptier who was equipped by WSP as part of intervention component two, while 20% (3/15) of intervention compounds reported using an intervention-supported pit-emptier. Adjusted for confounders, intervention compounds were 3.8 times (95% CI: 1.4, 10) more likely to have used a hygienic emptier in the previous year compared to control compounds.

Most hygienic emptying in control compounds occurred in a subset of those with pour-flush systems; at the 74 control compounds which reported emptying in the previous year, 36% (8/22) of those with pour-flush systems reported hygienic emptying compared to 3.8% (2/52) of those with dry pit latrines. For control compounds with pour-flush technology (n = 22) and intervention compounds (n = 15) that had emptied their sanitation system in the previous year, we used the unadjusted *Somer's d* statistic to characterize the association between intervention status and hygienic pit-emptying. We found a positive correlation between the presence of the intervention and hygienic pit-emptying in the previous year (d = 0.36, 95% CI: 0.06, 0.65).

Similarly, at compounds where an on-site sanitation system was emptied in the previous year, intervention compounds reported their fecal sludge was taken by the pit-emptier to a WWTP or transported away by a vehicle to an unknown location more frequently than control compounds (Text A.5); 2.7% of control compounds (2/74) reported their fecal sludge was transported away after emptying compared to 40% of intervention compounds (6/15). Adjusted for potential confounders, fecal sludge was 30 times more likely (95% CI: 3.3, 270) to have been transported away from intervention compounds compared to controls. In addition, intervention and control compounds that used mechanized emptying reported their waste was transported to a WWTP or away (47%, [8/17]) more frequently than compounds that used manual emptying (0%, 0/72) (Table 2).

**Table 2:** Locations where fecal sludge was deposited.

Reported destination of fecal sludge after emptying at compounds who emptied systems in the previous year (priority respondent)	Control compounds	Intervention compounds	Compounds that used mechanized emptying	Compounds that used manual emptying
Transported to WWTP	1.4% (1/74)	6.7% (1/15)	12% (2/17)	0% (0/72)
Transported away from compound to an unknown location	1.4% (1/74)	33% (5/15)	35% (6/17)	0% (0/72)
Buried on-site	77% (57/74)	27% (4/15)	0% (0/17)	85% (61/72)
Buried outside the compound	2.7% (2/74)	0% (0/15)	0% (0/17)	2.8% (2/72)
Dumped outside the compound	0% (0/74)	0% (0/15)	0% (0/17)	0% (0/72
Unsure	18% (13/74)	33% (5/15)	53% (9/17)	13% (9/72)

Among compounds that emptied in the previous year, reported visits by WSUP for any reason and visits to discuss emptying occurred more frequently at intervention compounds compared to controls (Table A2). At the subset of compounds that emptied hygienically in the previous year, a visit by WSUP for any reason was also more common at intervention compounds (82%, [9/11]) compared to controls (30%, [3/10]) and intervention compounds more often reported that WSUP discussed emptying during the visit (46%, [5/11]) than controls (30%, [3/10]) (Table A2).

Respondents reported various methods for deciding when to desludge (Table A3). Intervention respondents most often cited smell (40%, [215/542]), control respondents with pour-flush systems most often cited a visual inspection of the fecal sludge level (32%, [58/180]), and control respondents without a pour-flush system were most often unsure how they would decide (46%, [152/327]) (Table A3). Nearly one in five intervention respondents (19%, [101/542]) and control respondents with pour-flush systems (18%, [33/180]) stated they would decide to empty their sanitation system once it was overflowing (Table A3). Despite being designed for emptying every two years, few intervention respondents (8.3%, [45/542]) stated they would empty their system based on time in use.

Over half of intervention respondents (58%, [315/542]) indicated they would use a hygienic pit-emptier next time their latrine needs emptying, compared to 18% (89/507) of respondents from control compounds (Table 3). Most control respondents which stated a preference for future hygienic emptying possessed pour flush systems (Table A4). Adjusted for potential confounders, respondents from intervention compounds were 3.1 (95% CI: 2.4, 4.0) times more likely to express the intention to use a hygienic emptier and 0.54 (95% CI: 0.41, 0.70) times as likely to intend to use an unhygienic emptier the next time their on-site sanitation system needs emptying compared to controls (Table 3). A one-quartile increase in household wealth was associated with a decreased stated preference for future hygienic emptying at intervention compounds (aRR: 0.93, 95% CI: 0.88, 0.99) but we found no apparent association with control compounds (aRR: 0.99, 95% CI: 0.85, 1.15).

**Table 3:** Responses to stated preference survey questions.

	All Survey Respondents			Household Respondents		Compound Leader	
	**Control (n)**	**Intervention (n)**	**aRR (95% CI)**	Control (n)	Intervention (n)	**Control (n)**	**Intervention (n)**
**Respondent intends to use a hygienic emptier next time their on-site sanitation system needs emptying**	18% (89/507)	58% (315/542)	3.1 (2.4, 4.0)	17% (63/378)	54% (216/399)	20% (26/129)	69% (99/143)
**Respondent intends to use an intervention pit-emptier (from component two) next time their on-site sanitation system needs emptying**	0.39% (2/507)	5.9% (32/542)	12 (2.8, 48)	0.53% (2/378)	5.3% (21/399)	0% (0/129)	7.7% (11/143)
**Respondent intends to use an unhygienic emptier next time their on-site sanitation system needs emptying**	34% (174/507)	18% (100/542)	0.54 (0.41, 0.70)	33% (123/378)	19% (75/399)	40% (51/129)	17% (25/143)

Among respondents with a stated preference for future unhygienic emptying, most cited cost as the reason they would not choose hygienic empty. Among the 34% (174/507) of control respondents who indicated they plan to unhygienically empty next time their sanitation system needs emptying, 91% (158/174) cited cost and 7% (12/174) cited access as their reason for not intending to use a hygienic emptier (Table A5). Similarly, among the 18% (100/542) of intervention respondents who indicated they plan to unhygienically empty next time their sanitation system needs emptying, 81% (81/100) cited cost and 17% (17/100) cited access for not intending to use a hygienic emptier (Table A5). Intervention and control respondents who cited cost as their reason for not intending to hygienically empty had similar poverty scores (mean = 33/81, median = 31/81) compared to respondents who did not cite cost (mean = 30/81, median = 28/81).

## 4 Discussion

As cities in LMICs continue to grow in population and density, there is an increasing need for safe and sustainable sequestration of human fecal waste and hygienic FSM (Un-Habitat, 2003). We assessed the impact of an intervention providing latrines with septic tanks and FSM services 24–36 months after delivery. Though the sample size for intervention compounds which reported emptying in the previous year was small, we found that the intervention was significantly associated with increased hygienic emptying of septic tanks, increased transportation of sludge to a WWTP or away from the living environment and increased stated intention to engage hygienic FSM services in the future.

The less frequent emptying observed at intervention compounds may have been due to the recent construction of the intervention and the size of the septic tanks which may require less frequent desludging than pit latrines in Maputo (Bauerl et al., 2016). The proportion of shared latrines to community sanitation blocks that emptied in the previous year was the same as their proportion overall, suggesting both filled at similar rates. Intervention sanitation facilities were built during 2015–2016 and interviews took place in 2018. As some control respondents moved into their compounds after the on-site sanitation system had been constructed, we were unable to accurately determine the year that control systems were built for comparison. Additionally, intervention systems were not built in areas with high water tables; most control compounds were in the same neighborhoods as intervention compounds, but water table level was not a factor in control compound selection (Knee et al., 2018). Infiltration of water from a high water table into control systems—which predominantly occurs during the rainy season in Maputo (Hawkins and Muximpua, 2015)—may have contributed to the observed increase in emptying frequency.

Interventions targeting intentions are often more successful at changing infrequent behaviors (e.g. emptying) compared to frequent behaviors (e.g. handwashing) (Webb and Sheeran, 2006). Previous work in Maputo suggested that knowledge about available services is one factor that influences intentions for emptying (Bauerl et al., 2016). Our results showed that compound visits by WSUP—including visits that discussed emptying—were more frequent at intervention compounds than controls. The reported visits may have contributed to the observed increase in hygienic emptying at intervention compounds.

Fecal sludge in pour-flush systems is more watery than dry pit latrines and therefore more conducive to mechanized emptying than pit latrines which can be covered over or require manual removal of thick sludge (Strande et al., 2014). As expected from pour flush systems, most hygienic emptying occurred at intervention compounds and control compounds with pour-flush sanitation technology; transport of fecal wastes to the WWTP or away from the compound only occurred following mechanized emptying. Additionally, a septic tank is a larger capital investment in the sub-surface infrastructure than a dry pit latrine, which incentivizes emptying over replacement. In unplanned settlements globally—where fecal sludge burial is increasingly unworkable—this suggests that upgrading pit latrines to pour-flush to septic tank systems, in the presence of affordable hygienic emptying, may be one way to increase the likelihood fecal wastes are safely managed. Though when space is available covering over old pits is an acceptable solution for safe management.

Our results suggest hygienic pit emptying remains unaffordable for many in Maputo's low-income neighborhoods; participants most often used cost to justify a stated preference for unhygienic emptying. Depending on the volume of fecal sludge removed and distance transported to the WWTP, hygienic mechanized emptying typically costs $25-$50 USD while unhygienic manually emptying costs $8-$17 USD (2015 data) (Hawkins and Muximpua, 2015); wages in Mozambique may be $60 a month or less (2017 data) (Frey, 2017; Gerety, 2018). Household wealth was not associated with previous hygienic emptying which may suggest that emptying prices were expensive for both poor and relatively wealthier households. Wealthier intervention respondents were less likely to state a preference for future hygienic emptying than poorer intervention respondents. This may suggest courtesy or hypothetical response bias from poorer respondents (Hausman, 2012). Alternatively, poorer intervention residents may have been less likely than relatively wealthier residents to know the higher costs associated with hygienic emptying and subsequently stated an increased preference for hygienic emptying.

Progress is ongoing in Maputo to subsidize emptying for low-income residents. The Maputo Municipal Council (MMC) approved a sanitation tariff in December 2016 which would tax Maputo residents’ water bills to generate money. Revenue generated may be used for sanitation improvements and to subsidize hygienic emptying for low-income residents (Assembleia Municipal de Maputo, 2017) A similar approach has already been implemented in eThekwini, South Africa, which offers free pit-emptying to households using pit latrines every five years (WIN-SA, 2011). The proposed tariff and potential subsidy in Maputo may help achieve affordable hygienic emptying for low-income residents.

Overflowing septic systems may result in a direct human exposure to fecal sludge, an issue of public health concern. A study in Bhutan found most building owners intended to wait until their septic tank was overflowing to initiate emptying (Halcrow et al., 2014). We found a lower stated intention to delay emptying. Despite a recommendation to empty every two years to avoid overflowing, few intervention compounds reported an intention to empty based on time in use. Control compounds with pour-flush systems most often reported they use a visual inspection of fecal levels to time their pit-emptying. Intervention designs used in the Maputo Sanitation project made visual inspection of feces level impossible without masonry tools. Affordable, scheduled emptying of pour-flush to septic tank systems may be necessary to reduce intermittent exposure risks due to overflowing systems, especially in low lying areas prone to flooding in the rainy season. Additionally, a design feature such as an access port could be added to pour flush to septic tank systems to enable visual inspection, however access ports should not be easily removed to ensure a barrier exists between fecal waste and the domestic environment.

Although our observations have been limited to just one setting, our results suggest in this and potentially in similar unplanned urban settlements, interventions that include subsidized on-site sanitation designed for hygienic emptying—accompanied by visits to promote hygienic emptying—may increase the likelihood of hygienic emptying compared to compounds which do not receive the intervention, including compounds that construct their own pour-flush systems (Jenkins et al., 2014). Compounds with pour-flush sanitation may not have built their system in a location accessible by hygienic emptying or of a design compatible with the equipment used in hygienic emptying. A 2015 study in Dar es Salaam, Tanzania found that poor design or site placement of sanitation systems often necessitated damage to or destruction of the sanitation system in order for emptying to occur (Jenkins et al., 2015). Educating residents of and masons working in low-income unplanned settlements about suitable site placement and design of sanitation infrastructure for hygienic emptying would be useful but may not be realistic in many LMICs.

Over a period of many years, infrequent hygienic emptying may cost less than frequent unhygienic emptying. Bauerl et al. reported in Maputo that the annualized cost for mechanical emptying of pour flush facilities was less than the annualized manual emptying costs for traditional latrines, but more than the annualized manual emptying costs for improved latrines (the annualized costs included the initial capital investment costs of the infrastructure). However, the decreased frequency of emptying at intervention compounds we observed may not have contributed to the increased use of hygienic emptying; the urban poor often base decisions on cash flow, not lifetime cost (Baharoglu and Kessides, 2001). The economic reality for the urban poor suggests a sanitation levy to subsidize emptying may be necessary for safe management of fecal wastes. Additionally, when excreta are adequately sequestered on-site, a reduced emptying frequency subsequently reduces opportunities for human exposure to fecal waste and for excreta to spread into the environment, though hygienic emptying still poses risks for environmental fecal contamination. Where physically permissible, upgrading of traditional and improved latrines to pour flush to septic tank systems may reduce environmental fecal contamination and downstream exposure risks to enteric pathogens simply from less frequent emptying, though some data suggests septic tanks may require emptying more often than pit latrines (Strande et al., 2014).

We reviewed the data provided to us by the local municipality to assess the prevalence of emptying activities by the intervention equipped pit-emptiers. Between August 2017 and February 2018 (excluding December 2017), the pit-emptiers accounted for 0.95% (112/11,831) of all truck visits to dispose of fecal sludge at the Infulene WWTP. Amongst trucks originating from intervention neighborhoods, the pit-emptiers accounted for 4.2% (28/667) of truck visits to the WWTP. Between August 2017 and February 2018, 25% (28/112) of the truck visits to the WWTP by the intervention pit-emptiers originated in intervention neighborhoods, suggesting the companies served low-income residents in the project area and residents outside the project area. Similar work by WSUP in Bangladesh demonstrated that for subsidized emptying companies a 70/30 mix of high-income and commercial customers to customers in low-income neighborhoods provided sufficient profit to encourage participation (Renouf and Drabble, 2018). To guarantee service to low-income neighborhoods, WSUP subsidized emptiers in Bangladesh can be fined if <30% of their customers do not live in low-income neighborhoods. Where the target market cannot support the full cost of emptying, and enough high-income residents exist to provide a cross-subsidy, a similar approach may be useful to provide hygienic emptying services in Maputo and other LMICs.

The population density of *Nhlamankulu* district is high; of the 11 neighborhoods in the *Nhlamankulu* District five have 15,000–20,000 inhabitants/km^2^ and six exceed 20,000 inhabitants/km^2^ (WaterAid, 2013). In this densely populated setting some compound residents did not state a preference for future hygienic emptying due to reported limited physical access by hygienic emptiers, which is common in informal settlements globally (Chowdhry and Kone, 2012). Pour flush systems produce a greater volume of fecal sludge that when emptied unhygienically, is more mobile than the sludge from dry pit latrines and may subsequently pose greater risks for environmental fecal contamination. For areas in low income settlements inaccessible to hygienic emptying, improving hygienic emptiers’ access to latrines through transportation infrastructure improvements (e.g. road widening) is crucial to safely manage fecal waste, especially during the expansion of piped water which was associated with an increase in pour flush systems in Maputo (Hawkins and Muximpua, 2015). Our results corroborate this previous observation in Maputo; greater access to tap water inside a compound correlated with an increased prevalence of pour-flush sanitation. Additionally, at high population densities simplified sewerage may have a lower annualized cost per capita than on-site sanitation (Sinnatamby et al., 1985). Piped sewers and wastewater treatment should remain long-term goals in such a setting.

### 4.1 Limitations

There are various limitations to the reliability and external validity of our results. The cross-sectional nature of our study can only identify associations, not causation. Respondents were asked to remember past events, which introduced the potential for recall bias. Survey questions included hypothetical questions about the future, which introduced the potential for hypothetical response bias (Hausman, 2012). Average compound wealth scores did not include the compound leader's wealth, which may have biased our results. Due to the nature of the intervention, study participants were not blinded to the intervention, which may have led to interviewer or courtesy bias. As the intervention was recently implemented, very few intervention compounds had previously emptied their on-site sanitation system, which led us to draw conclusions about emptying practices from a small subset of all intervention compounds. Additionally, our findings represent emptying in one area of Maputo and may not be applicable to other cities in LMICs.

## 5 Conclusion

FSM is a critical area of urban health and development with few existing data; demographic trends indicate the population living in low-income unplanned settlements is increasing. Our results indicate that the provision of on-site sanitation systems consisting of pour-flush latrines to septic tanks that were accompanied by implementer visits to recipients, and where safe emptying services were available, increased the use of such services but were unaffordable for many users.

## Data availability statement

Data will be publicly available at https://osf.io/p5shk/ upon acceptance of the manuscript.

## Financial disclosure

This study was funded by the Bill and Melinda Gates Foundation (www.gatesfoundation.org) grant OPP1137224 to OC and JB. The funders had no role in study design, data collection and analysis, decision to publish, or preparation of the manuscript.

## Figures and Tables

**Figure:**
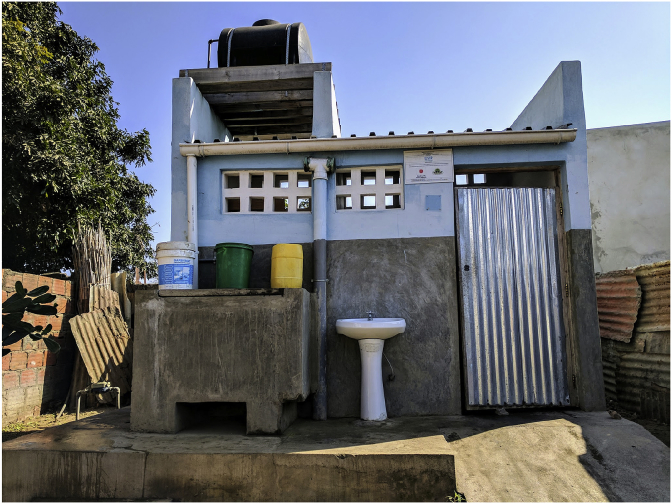


**Figure:**
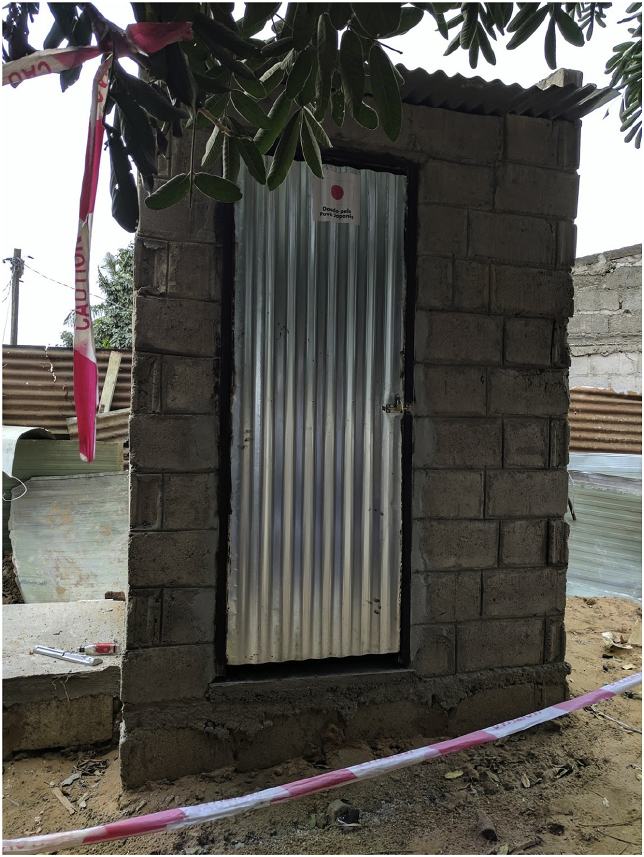


**Figure:**
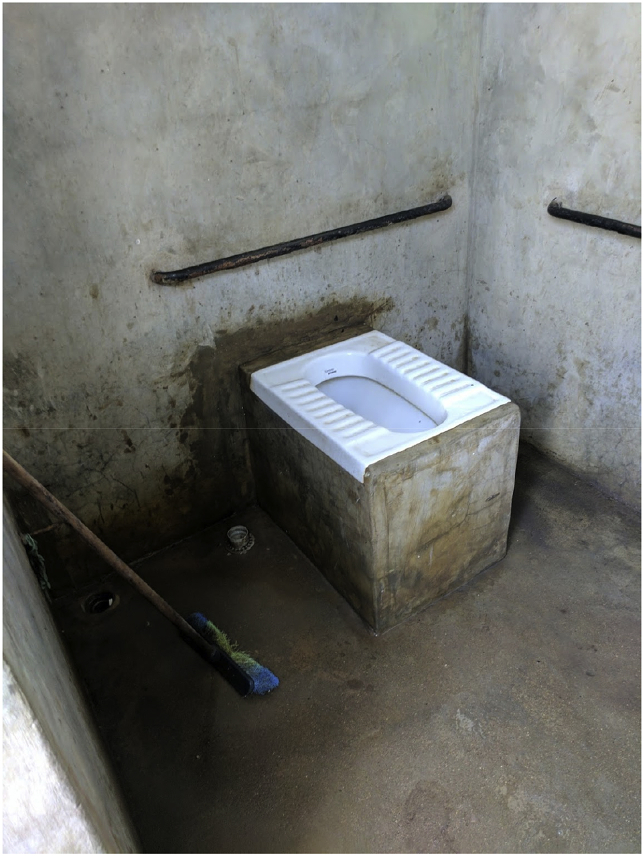


**Table A.1:** Water access and availability

Water Source	Control				Intervention	
	MapSan respondent	non-MapSan respondent	Pour flush sanitation	Not pour flush sanitation	MapSan respondent	non-MapSan respondent
Tap in the home	24% (43/179)	15% (30/199)	19% (27/140)	19% (46/238)	34% (60/176)	24% (54/223)
Tap outside the home	36% (65/179)	40% (79/199)	50% (70/140)	31% (74/238)	40% (70/176)	39% (87/223)
Neighbor's tap	37% (66/179)	43% (85/199)	31% (43/140)	45% (108/238)	25% (44/176)	35% (77/223)
Public tap	2.8% (5/179)	2.5% (5/199)	0% (0/140)	4.2% (10/238)	0.57% (1/176)	2.2% (5/223)
Protected spring	0% (0/179)	0% (0/199)	0% (0/140)	0% (0/238)	0.57% (1/176)	0% (0/223)

**Water availability**						
<1 h	0% (0/179)	0% (0/199)	0% (0/140)	0% (0/238)	0% (0/176)	0% (0/223)
1–3 h	15% (26/179)	15% (29/199)	12% (17/140)	16% (38/238)	15% (27/176)	14% (31/223)
4–6 h	36% (65/179)	37% (74/199)	38% (53/140)	36% (86/238)	39% (69/176)	43% (95/223)
7–8 h	33% (59/179)	36% (72/199)	34% (48/140)	35% (83/238)	32% (57/176)	29% (64/223)
>8 h	16% (29/179)	12% (24/199)	16% (22/140)	13% (31/238)	13% (23/176)	15% (33/223)

**Table A.2:** Reponses to whether WSUP visited the respondent and discussed pit-emptying (priority respondent).

	Control		Intervention	
	Emptied unhygienically in the past year	Emptied hygienically in the past year	Emptied unhygienically in the past year	Emptied hygienically in the past year
Participant ever received a WSUP visit and emptying was discussed	20% (13/64)	30% (3/10)	50% (2/4)	46% (5/9)
Participant ever received a WSUP visit, but emptying was not discussed	13% (8/64)	0% (0/10)	0% (0/4)	36% (4/9)
Participant did not recall a WSUP visit	53% (34/64)	60% (6/10)	25% (1/4)	18% (2/9)
Unsure	14% (9/64)	10% (1/10)	25% (1/4)	0% (0/9)

**Table A.3:** How will residents decide the sanitation system requires emptying (all respondents).

Next time a sanitation structure is emptied, how will people in the compound determine the sanitation structure needs to be emptied?	Control latrine (with or without a slab)	Control with pour-flush technology	All control compounds	Intervention compounds
**Smell**	7.0% (23/327)	22% (40/180)	12% (63/507)	40% (215/542)
**Visual inspection of fecal sludge level**	38% (124/327)	32% (58/180)	36% (182/507)	19% (101/542)
**A certain amount of time has passed**	2.1% (7/327)	14% (25/180)	6.3% (32/507)	8.3% (45/542)
**The sanitation structure is overflowing**	6.1% (20/327)	18% (33/180)	10% (53/507)	19% (102/542)
**The sanitation structure is damaged**	0% (0/327)	0% (0/180)	0% (0/507)	55% (3/542)
**Other**	31% (1/327)	2.8% (5/180)	1.2% (6/507)	55% (3/542)
**Unsure**	46% (152/327)	11% (19/180)	34% (171/507)	13% (73/542)

**Table A.4:** Intentions for future emptying (all respondents).

Reported intention for next time the sanitation system requires emptying	Control latrine (with or without a slab)	Control with pour-flush technology	All control compounds	Intervention compounds
**Intend to cover the current pit and open a new pit in the compound**	45% (140/327)	5.5% (10/180)	30% (150/507)	0% (2/542)
**Intend to cover the current pit and use neighbor's latrine**	0% (0/327)	0% (0/180)	0% (0/507)	0% (1/542)
**Intend to empty by household member**	6.7% (22/327)	7.2% (13/180)	7% (35/507)	1% (6/542)
**Intend to empty by community member**	34% (99/327)	22% (40/180)	27% (139/507)	17% (94/542)
**Intend to empty by a business or non-governmental organization**	3.7% (12/327)	43% (77/180)	18% (89/507)	58% (315/542)
**Intend to replace the entire sanitation system**	2.1% (7/327)	0% (0/180)	1% (7/507)	0% (0/542)
**Unsure**	14 (47/327)	22% (40/180)	17% (87/507)	23% (124/542)

**Table A.5:** Reasons for not stating a preference for hygienic emptying among those who stated a preference for future unhygienic emptying.

Respondent	Intervention				Control			
	MapSan	Non-MapSan	Compound Leader	All	MapSan	Non-MapSan	Compound Leader	All
Cost	74% (32/43)	91% (29/32)	80% (20/25)	81% (81/100)	92% (54/59)	94% (60/64)	86% (44/51)	91% (158/174)
Access	23% (10/43)	6% (2/32)	20% (5/25)	17% (17/100)	5% (3/59)	5% (3/64)	12% (6/51)	7% (12/174)
Other*	2% (1/43)	3% (1/32)	0% (0/25)	2% (2/100)	3% (2/59)	2% (1/64)	2% (1/51)	2% (4/174)

*Other responses included unsure (n = 4), “because all my neighbors do that [unhygienically empty]” (n = 1), and “I don't know anyone [who does hygienic emptying]” (n = 1).

## Appendices

### Text A.1: Detailed description of sanitation intervention

WSUP initially built 250 shared toilets and 50 community sanitation blocks, but due to the depreciation of the Metical (Mozambican currency) in 2016–2017, WSUP built 150 additional shared toilets in the project area. Shared latrines became the property of the residents and included a toilet, superstructure, septic tank, and a lined infiltration pit. Community sanitation blocks officially remained the property of the municipality and included the same infrastructure as a shared latrine, but contained multiple toilets (one toilet per twenty people), a new piped water connection with a water storage tank, sink pedestal for handwashing (no running water but the drain was connected to the septic tank), rainwater harvesting tank, cement laundry basin, and community sanitation blocks with ≥60 residents received a urinal on an external wall of the structure which drained to the septic tank. Compound residents that received community sanitation blocks formed sanitation management committees, which were responsible for maintaining the sanitation infrastructure. The septic tanks in the shared latrines and community sanitation blocks were sized according to the number of users and were designed to be emptied every two years (assuming 40 L accumulation per person per year). All intervention septic tanks contained an access port for hygienic emptying, but the ports were sealed shut and did not enable easy visual inspection of fecal sludge levels.

### Text A.2: Method for identifying non-MapSan trial respondent

In recognition that the caregivers of children enrolled in the MapSan trial (R1) represented a relatively homogenous group (predominantly women of reproductive age) a second compound respondent was included in the survey sample (R2). To select R2, enumerators identified the third household on the right of the entrance to the compound (providing this was not the household of R1). R2 could be any gender or age, so long as they were over 18 and had lived on the compound for more than 30 days. If there was no one available in the identified household, or the respondent refused to participate, the enumerator would identify the next third house on the right.

### Text A.3: Wealth calculations

Compound leaders were typically the longest resident in each compound. The poverty scorecard we used score is less an indicator of current income but better reflects previous cash flow to purchase durable goods, suggesting compound leaders may systematically score higher than other compound residents.

In addition, the surveys we conducted typically lasted 45–60 min. We had concerns that our previous surveys among this cohort--compounded by this survey's length—may have resulted in survey fatigue. To minimize the potential for survey fatigue we focused questions based on respondent type. We asked household respondents survey questions that focused on household level info but did not include some household level questions in surveys of compound leaders. As a result, we did not calculate household wealth for compound leaders, but instead took the average of the household wealth scores from household respondents as the representative household wealth score for each compound.

### Text A.4: Details regarding compounds that did not meet eligibility requirements

We deemed 48 control respondents from 30 control compounds ineligible for the study as the intervention latrine infrastructure was present (additional construction explained in S1 Text). We deemed 15 intervention respondents from nine intervention compounds ineligible due to the absence of the intervention latrine infrastructure at the intervention compounds.

### Text A.5: Detailed description of fecal sludge transport

We asked survey respondents where their fecal sludge was disposed last time they emptied their sanitation system. As survey respondents had only witnessed pit-emptiers while they were on-site, they were likely able to recall if their fecal sludge was dumped or buried in or near their compound. However, if fecal sludge was transported away by a truck or tractor, they likely did not have certainty regarding the final disposal site for their fecal sludge.

In addition to compound interviews, we conducted structured in-depth interviews on FSM topics with the staff that took part in component two of the intervention and were equipped to provide pit-emptying services. We posed questions on the status and characteristics of the CBOs’ emptying activities, their experience working with WSP, and their difficulties and successes as pit-emptiers in Maputo. Of the eight formal operators trained by WSUP as part of the JSDF funded intervention, we met with the four who had continued to service clients after the pilot period ended in 2015. We were unable to contact the four operators who had ceased servicing clients.

Due to the statements by the four pit-emptying organizations surveyed that they never illegal dumped fecal sludge, the illegality of dumping in Maputo, the close proximity of *Nhlamankulu* District to the WWTP (typically a 7–25 min drive depending on intra-district location) and the potential for reputational risk to hygienic emptying companies who engage in illegal dumping, we inferred that respondents who stated their fecal sludge was transported away to an unknown location was indicative of hygienic transport to the local WWTP.
